# Psychometric Evaluation of the Parkinson's Disease Activities of Daily Living Scale

**DOI:** 10.1155/2017/4151738

**Published:** 2017-09-25

**Authors:** Stina B. Jonasson, Peter Hagell, Gun-Marie Hariz, Susanne Iwarsson, Maria H. Nilsson

**Affiliations:** ^1^Department of Health Sciences, Faculty of Medicine, Lund University, Lund, Sweden; ^2^Department of Neurology and Rehabilitation, Skåne University Hospital, Lund, Sweden; ^3^The PRO-CARE Group, School of Health and Society, Kristianstad University, Kristianstad, Sweden; ^4^Department of Community Medicine and Rehabilitation, Occupational Therapy, Umeå University, Umeå, Sweden; ^5^Memory Clinic, Skåne University Hospital, Malmö, Sweden

## Abstract

**Objective:**

To evaluate a set of psychometric properties (i.e., data completeness, targeting, and external construct validity) of the Parkinson's disease Activities of Daily Living Scale (PADLS) in people with Parkinson's disease (PD). Specific attention was paid to the association between PADLS and PD severity, according to the Hoehn & Yahr (H&Y) staging.

**Methods:**

The sample included 251 persons with PD (mean age 70 [SD 9] years). Data collection comprised a self-administered postal survey, structured interviews, and clinical assessments at home visits.

**Results:**

Data completeness was 99.6% and the mean PADLS score was 2.1. Floor and ceiling effects were 22% and 2%, respectively. PADLS scores were more strongly associated (*r*_*s*_ > 0.5) with perceived functional independence, ADL dependency, walking difficulties, and self-rated PD severity than with variables such as PD duration and cognitive function (*r*_*s*_ < 0.5). PADLS scores differed across H&Y stages (Kruskal-Wallis test, *p* < 0.001). Those in H&Y stages IV-V had more ADL disability than those in stage III (Mann–Whitney *U* test, *p* < 0.001), whereas there were no significant differences between the other stages.

**Conclusion:**

PADLS revealed excellent data completeness, acceptable targeting, and external construct validity. It seems to be well suited as a rough estimate of ADL disability in people with PD.

## 1. Introduction

The ability to perform activities of daily living (ADL) is essential for independent living. ADL includes activities such as feeding, dressing, bathing, cooking, cleaning, and shopping. People with Parkinson's disease (PD) often experience ADL limitations already early during the disease course [1]. Poor ADL performance may result in dependence and is negatively associated with health-related quality of life [2]. Thus, adequate assessments of ADL are important to be able to monitor ADL performance throughout the course of disease in order to provide optimal treatment, care, and rehabilitation for people with PD.

There are various ways of assessing ADL. The assessment can be based on observations of actual ADL performance, interviews, or self-ratings. Assessments can address difficulties in performing ADL, dependence on assistance from others and from assistive devices, or a combination of both [3]. Several ADL rating scales have been psychometrically tested and recommended for use with people with PD [3]. These rating scales typically include around ten items and have been shown to be highly associated with walking difficulties (*r*_*s*_ 0.74–0.86) [4] and physical functioning (*r* 0.63–0.77) [5, 6]. Lower associations have been reported between ADL disability and depressive symptoms (*r*_*s*_ 0.45–0.61) [7, 8], general health (*r* 0.42–0.47) [5], PD duration (*r*_*s*_ 0.40) [7], cognitive function (*r*_*s*_ 0.18–0.23) [8], and age (*r*_*s*_ 0.16) [7], respectively. The association between ADL disability and PD motor symptoms has shown large variations among studies (*r* 0.37–0.68; *r*_*s*_ 0.52–0.87) [5–7, 9]. Large variations apply also for the association between ADL disability and PD severity (*r* 0.54–0.59; *r*_*s*_ 0.36–0.83) [5–9].

The Parkinson's disease Activities of Daily Living Scale (PADLS) is a single-item self-reported rating scale targeting ADL in people with PD [10]. Since its development in 2001, the PADLS has been used in various PD studies; see, for example, [11–15]. To the best of our knowledge, its psychometric properties have only been reported in the original publication [10] and in a conference abstract [16]. These studies [10, 16] reported test-retest reliability (weighted Kappa 0.70;* r* 0.89) and external construct validity in terms of associations with, for example, motor symptoms (*r* 0.65; *r*_*s*_ 0.55), complications of PD therapy (*r*_*s*_ 0.56), depressive symptoms (*r* 0.43), PD duration (*r* 0.39; *r*_*s*_ 0.32), and frequency of social activities (*r* 0.02–0.44). A recent review listed PADLS as a “suggested” rating scale for assessment of ADL in people with PD, and it was argued that more psychometric studies are needed before PADLS can be classified as “recommended” [3]. The review also noted a lack of studies regarding the association between PADLS and the Hoehn & Yahr staging (H&Y, i.e., a classification of PD severity [17]) and the Unified Parkinson's Disease Rating Scale (UPDRS, which assesses PD signs and symptoms [18]).

Thus, this study aimed to evaluate a set of psychometric properties (i.e., data completeness, targeting, and external construct validity) of PADLS scores in people with PD. Specific attention was paid to the association between PADLS and PD severity according to H&Y.

## 2. Materials and Methods

We utilized cross-sectional baseline data of the project “Home and Health in People Ageing with PD.” Details regarding the project design and methods have been published elsewhere [19]. The project was conducted in accordance with the Helsinki Declaration and was approved by the Regional Ethical Review Board in Lund, Sweden (number 2012/558). All participants gave their written informed consent.

### 2.1. Participants and Recruitment

Participants were recruited from three hospitals in Skåne County, Sweden. A detailed flow chart of the recruitment procedure has been published [20]. A total of 653 persons met the inclusion criterion of a PD diagnosis (ICD-10: G20.9) since at least one year. Of those, 158 were excluded due to difficulties in understanding or speaking Swedish (*n* = 10), severe cognitive difficulties (*n* = 91), or other reasons that made them unable to give informed consent or take part in the majority of the data collection (e.g., hallucinations or a recent stroke, *n* = 57). Fifty-eight persons were excluded since they lived outside Skåne County. The remaining 437 persons were invited to participate, but 22 of those were unreachable and two had a revised diagnosis. Out of the remaining 413 participants, 157 (38%) declined. Another five persons were excluded since they had not responded to the PADLS by themselves or not responded within two months from the home visit (part of the data collection) or due to extensive missing data. This resulted in a final study sample of 251 participants (mean age 70 [SD 9] years; 39% women). Further participant characteristics are presented in Table 1.

### 2.2. Data Collection

The data collection is comprised of a self-administered postal survey and a subsequent home visit, which included interview-administered questionnaires and questions as well as clinical assessments. The home visits were conducted by two registered occupational therapists who had undergone project-specific training. More details regarding the procedure have been described elsewhere [19].

The self-administered postal survey included the PADLS, a single-item self-reported rating scale that addresses perceived ADL difficulties and dependence on others as well as on assistive devices during various ADL [10]. Respondents are instructed to rate how their PD has affected their day-to-day activities during the past month according to five response categories ranging from 1 (no difficulties with day-to-day activities) to 5 (extreme difficulties with day-to-day activities), but each response option also has a more detailed description. For example, “2: mild difficulties” includes the following description: “Slowness with some aspects of housework, gardening or shopping. Able to dress and manage personal hygiene completely independently but rate is slower.” PADLS scores have also been dichotomized into “not needing help from others in daily activities” versus “needing help” (PADLS 1-2 versus 3–5) [21].

In addition, the postal survey included a question on self-rated general health (possible item score 1–5, higher = worse) [22] and the Generic Walk-12 (Walk-12G), which assesses perceived walking difficulties in everyday life (possible total score 0–42, higher = worse) [23].

The structured interview during the home visit included a question on PD duration and three study-specific questions targeting social activities. The latter were self-rated by asking about the frequency of visiting/receiving visits from friends/family (almost never; once or twice a year, once or twice a month, once or twice a week, or every day; scored 1–5, resp.). Moreover, depressive symptoms were self-rated using the Geriatric Depression Scale (GDS-15; possible total score 0–15, higher = worse) [24]. Perceived functional independence was assessed according to an item from the Neuropsychological Aging Inventory (possible item score 0–10, higher = better) [25]. Finally, ADL dependency was assessed using the ADL Staircase [26]. Based on the internationally well-known and widely used Katz' ADL Index [27], the ADL Staircase is a conceptually and theoretically sound instrument supported by research demonstrating reliability and validity [28, 29] as well as methodological considerations for use in different populations [30, 31]. Dependence in nine ADL items is rated based on a combination of interview and observation (possible total score 0–9, higher = worse) [26].

Motor symptoms were clinically assessed according to the UPDRS part III (possible total score 0–108, higher = worse), whereas complications of PD therapy were assessed according to the UPDRS part IV (possible total score 0–23, higher = worse) [18]. Cognitive function was assessed by using the Montreal Cognitive Assessment (MoCA; possible total score 0–30, higher = better) [32]. PD severity was assessed using the H&Y (stages I–V, higher = worse) [17]. In addition, the participants rated their overall PD severity as either mild, moderate, or severe (scored 1–3, resp.). Assessments at the home visits were conducted at a time point when the participant reported feeling at their best.

### 2.3. Data Analyses

Statistical analyses were performed in IBM SPSS Statistics, version 24. Analyses included data completeness, targeting, and external construct validity of the PADLS. Two-tailed *p* values were used and the level of statistical significance was set to *p* < 0.05.

#### 2.3.1. Data Completeness

Data completeness refers to the degree to which a rating scale is completed [33, 34] and was calculated as the percentage of participants who responded to the PADLS. A maximum of 10% missing data has been suggested as a limit for acceptable data completeness [35].

#### 2.3.2. Targeting

Targeting refers to the scale's ability to mirror the levels of the targeted variable (e.g., ADL disability) in the study sample [33]. The mean score of a well-targeted rating scale should be close to the scale's midpoint and scores should range the full span of possible scale scores [34]. Skewness should be less than ±1 [34] and floor and ceiling effects should not exceed 15–20% [33, 34, 36]. That is, less than 15–20% of the study sample should score 1 or 5 on the PADLS, respectively.

#### 2.3.3. External Construct Validity

External construct validity of a rating scale is supported when scores are more strongly associated with related constructs and more weakly associated with nonrelated constructs [37]. In this study, associations between PADLS and other scores were explored by Spearman's correlation coefficients (*r*_*s*_). The hypotheses were based on clinical reasoning and previous studies regarding associations between ADL and other variables [4–10, 16]. The associations (*r*_*s*_) between PADLS and walking difficulties in daily life, perceived functional independence, self-rated PD severity, ADL dependency, motor symptoms, and complications of PD therapy were anticipated to be >0.5 [4–7, 9, 10, 16]. The associations between PADLS and depressive symptoms as well as general health were anticipated to be around 0.5 [5, 7, 8, 10]. The associations between PADLS and PD duration, age, cognitive function, and frequency of social activities were anticipated to be <0.5 [5, 7, 8, 10, 16].

Kruskal-Wallis and Mann–Whitney *U* tests were used to explore whether PADLS scores differed between H&Y stages. H&Y stages IV and V were merged due to few participants in H&Y stage V (*n* = 6).

## 3. Results

All but one participant responded to the PADLS, resulting in 99.6% data completeness. The score distribution is presented in Table 2. The mean score was 2.1 and scale scores ranged the full span (i.e., 1–5). Fifty-four participants chose the lowest (“best”) response option (22% floor effect) whereas six participants chose the highest (“worst”) response option (2% ceiling effect).

PADLS scores correlated >0.5 with walking difficulties in daily life, perceived functional independence, self-rated PD severity, and ADL dependency. The associations between PADLS scores and other studied variables were weaker (Table 3). The Kruskal-Wallis test showed that PADLS scores differed across H&Y stages (*p* < 0.001). Specifically, those in H&Y stages IV and V had higher PADLS scores than those in H&Y stage III (Mann–Whitney *U* test, *p* < 0.001), whereas there were no significant differences between the other H&Y stages (Table 4).

## 4. Discussion

This study confirmed that the PADLS has satisfactory psychometric properties for use in the PD population. The PADLS revealed excellent data completeness; only one participant left the form blank. This indicates that the scale is easy to understand and perceived as relevant [38] by people with PD. The finding probably reflects the single-item nature of the scale, which might favor data completeness. Targeting was generally acceptable, with small ceiling effects. However, floor effects were above the recommended level [33, 34, 36]. Small floor and ceiling effects are desirable in order to enable separation of people and detect changes [33]. More than one-fifth scored the lowest possible and reported no difficulties with day-to-day activities. This floor effect may purely mirror the PD severity of the present sample. That is, 85 participants self-rated their PD as mild and 50 were classified as H&Y stage I. With such a high prevalence of mild PD severity it is not surprising that also ADL disabilities were rated as low or nonexisting. On the other hand, a previous study [1] reported that restrictions in ADL are often seen early during the disease. Already at their first visit to a neurological centre, those later diagnosed with PD had more ADL disabilities than healthy age-matched controls [1]. Although the PADLS showed generally satisfactory psychometric properties, its single-item nature makes it a coarse indicator unsuitable as an outcome measure due to the uncertainty associated with such scores. Notably, floor effects indicate that it is especially important to complement this scale with a more detailed ADL assessment for people with mild PD-symptoms.

External construct validity of the PADLS was generally supported by largely expected associations with other variables. However, the associations with motor symptoms and complications of PD therapy were lower than expected. In comparison to the present sample, the participant characteristics in previous studies [5, 6, 9, 10, 16] show no clear patterns that could explain our relatively weak association between, for example, ADL disability and motor symptoms. However, previous studies have shown large variations in association between motor symptoms and ADL disability, and our findings are within the range of previous studies [5–7, 9, 10, 16].

Indeed, varying results in previous studies imply a challenge when stating a priori hypotheses, which is essential for the exploration of a scale's external construct validity [37]. We do find our hypothesis of the association between ADL disabilities and motor symptoms reasonable, as several items in UPDRS part III (i.e., motor symptoms) do capture disabilities. It should be kept in mind that ADL is a complex phenomenon, affected by environmental characteristics and prerequisites as well as by the use of various assistive devices. Moreover, existing ADL assessments cover different activities, and the majority do not take individual or subgroup specific activity preferences and patterns into consideration. All considered, ADL rating scales take different aspects into account and include different activities, making the definition of a priori hypotheses a delicate matter.

Although PADLS scores increased with increasing H&Y stages, the differences across these stages were small. The finding that PADLS scores differed only between H&Y stage III versus stages IV and V is not surprising. That is, the definition of H&Y stage III states that “patients are still physically capable of leading independent lives” whereas stages IV-V define a “severely disabling” PD [17].

### 4.1. Limitations

Data completeness might have been affected by the study design. That is, our data collectors were instructed to screen all self-administered ratings at the subsequent home visit. In case of missing values, the data collectors were instructed to ask the participants to add responses. However, another psychometric study based on the same data collection did report missing values that were close to those collected in another sample not using the same procedure [39].

One could argue that using polychoric and polyserial correlation coefficients would have been a theoretically better choice than *r*_*s*_ when studying the external construct validity of the PADLS. This is since *r*_*s*_ and *r* tend to attenuate estimated correlations between ordinal data [40, 41], such as the PADLS. Since previous studies did not use polychoric or polyserial correlations [4–10, 16], we used methods similar to those used before in order to enhance comparability with previous studies. However, reanalyses using polychoric correlations yielded generally somewhat stronger coefficients, as expected (data available on request). In addition, this study does not cover all psychometric aspects. For example, test-retest reliability was not evaluated.

Our decision to use the ADL Staircase [26] and not the UPDRS part II [18] to study external construct validity in terms of ADL deserves a comment since the latter is commonly used in PD research. Given the complexity of the phenomenon at target (i.e., ADL), our ambition was to use data collected with an ADL rating scale based on conceptual and theoretical underpinnings. As such, ADL is not disease specific but a generic human phenomenon. Although UPDRS part II is recommended as a disability instrument [3], it contains items that are not conceptually related to the ADL construct [42]. Recently the Movement Disorder Society task force documented this as a major drawback as “the 13 items of the UPDRS-ADL do not all assess disability, with 7 of 13 assessing impairments, not functional status (speech, salivation, swallowing, falling, freezing, tremor, and sensory complaints)” [3]. While we consider choosing the ADL Staircase a methodological strength, it should be kept in mind that the comparability with PD studies that used UPDRS part II is limited.

It needs to be emphasized that the PADLS is a single-item rating scale and as such, it only gives a rough, global estimate of a person's ADL disabilities. Still, it should be noted that although the abbreviated response categories are phrased, for example, “mild difficulties in day-to-day activities,” the PADLS captures both perceived difficulties, dependence on others and on assistive devices during ADL performance. Moreover, it includes both personal and instrumental ADL. As such, the PADLS can be useful in clinical practice as well as in research for purposes of providing a crude categorization of levels of ADL disabilities. However, a more comprehensive rating scale is needed if a more thorough ADL assessment is warranted, and especially so for those with less severe ADL disabilities. This is in agreement with recommendations from the developers of the PADLS, who stated that the scale is not suitable for use in isolation but should be considered a complement to existing scales [10].

## 5. Conclusions

We found the self-reported, single-item rating scale PADLS to yield scores with excellent data completeness, acceptable targeting, and external construct validity. The PADLS seems to be well suited for providing a rough indicator of ADL disability in people with PD. As psychometric testing is a continuous process, further studies focusing on additional aspects, such as responsiveness, minimal important difference, and response category functioning, are warranted.

## Figures and Tables

**Table 1:** Participants' characteristics (*n* = 243–251, depending on missing data).

Characteristics	Median (first-third quartile) unless otherwise stated
Sex (women), *n* (%)	99 (39)
Age (years), mean (SD), min–max	70 (9), 45–93
Parkinson duration (years)	8 (5–13)
Parkinson severity (Hoehn & Yahr)^1^	3 (2-3)
Parkinson severity (self-rated), *n* (%)	
Mild	85 (34)
Moderate	116 (46)
Severe	49 (20)
Motor symptoms (UPDRS part III)^2^	30 (22–39)
Complications of therapy (UPDRS part IV)^3^	4 (2–7)
Self-rated general health (RAND-36)^1^	3 (3-4)
ADL dependency (ADL staircase)^4^	1 (0–3)
Perceived functional independence^5^	9 (7–10)
Walking difficulties in daily life (Generic Walk-12)^6^	14 (7–24)
Depressive symptoms (Geriatric Depression Scale)^7^	2 (1–4)
Cognitive function (Montreal Cognitive Assessment)^8^	26 (22–28)

UPDRS = Unified Parkinson's Disease Rating Scale; ADL = activities of daily living; possible score ranges and directions: ^1^1–5, higher = worse; ^2^0–108, higher = worse; ^3^0–23, higher = worse; ^4^0–9, higher = worse; ^5^0–10, higher = better; ^6^0–42, higher = worse; ^7^0–15, higher = worse; ^8^0–30, higher = better.

**Table 2:** Score distribution of the Parkinson's disease Activities of Daily Living Scale (PADLS).

PADLS response options (abbreviated)	*n *(%)^1^
(1) No difficulties with day-to-day activities	54 (22)
(2) Mild difficulties with day-to-day activities	130 (52)
(3) Moderate difficulties with day-to-day activities	49 (20)
(4) High levels of difficulties with day-to-day activities	11 (4)
(5) Extreme difficulties with day-to-day activities	6 (2)

Mean score (SD)	2.1 (0.9)
Median (first-third quartile)	2 (2-3)
Skewness (SE)	0.94 (0.15)
Floor/ceiling effect	21.6%/2.4%

^1^One person did not respond to the PADLS, resulting in *n *= 250.

**Table 3:** Spearman correlations (*r*_*s*_) between Parkinson's disease Activities of Daily Living Scale (PADLS) scores and other variables.

Variable	*r*_*s*_	*p* value
Walking difficulties in daily life (Generic Walk-12)	0.66	<0.001
Perceived functional independence	−0.62	<0.001
Parkinson severity (self-rated)	0.59	<0.001
ADL dependency (ADL staircase)	0.54	<0.001
Depressive symptoms (Geriatric Depression Scale)	0.40	<0.001
Motor symptoms (UPDRS part III)	0.39	<0.001
Self-rated general health (RAND-36)	0.38	<0.001
Parkinson duration	0.33	<0.001
Complications of therapy (UPDRS part IV)	0.29	<0.001
Age	0.23	<0.001
Cognitive function (Montreal Cognitive Assessment)	−0.19	0.002
Frequency of social activities: visits family	−0.10	0.104
Frequency of social activities: visits friends	−0.06	0.332
Frequency of social activities: receives visits from family or friends	0.02	0.774

Higher scores are worse for all variables, except for perceived functional independence and cognitive function (higher = better) and frequency of social activities (higher = more social activities). This implies that more, for example, walking difficulties and less, for example, functional independence are associated with more ADL disability (positive and negative correlation coefficients, resp.); ADL = activities of daily living; UPDRS = Unified Parkinson's Disease Rating Scale; *n* = 243–250, depending on missing data.

**Table 4:** Descriptive data of Parkinson's disease Activities of Daily Living Scale (PADLS) scores across Hoehn & Yahr stages.

Hoehn & Yahr		PADLS, median (first-third quartile)	Need help from others in daily activities, *n *(%)^1^
Stage	*n* (%)		
I	50 (20)	2 (1-2)	5 (10%)
II	72 (29)	2 (1-2)	11 (15%)
III	67 (27)	2 (2-2)	12 (18%)
IV-V	61 (24)	3 (2–3.5)	38 (62%)

^1^PADLS dichotomized: those who scored >2 were classified as needing help from others.

## References

[1] Hariz G.-M., Forsgren L. (2011). Activities of daily living and quality of life in persons with newly diagnosed Parkinson's disease according to subtype of disease, and in comparison to healthy controls. *Acta Neurologica Scandinavica*.

[2] Lawrence B. J., Gasson N., Kane R., Bucks R. S., Loftus A. M. (2014). Activities of daily living, depression, and quality of life in Parkinson's disease. *PLoS ONE*.

[3] Shulman L. M., Armstrong M., Ellis T. (2016). Disability Rating Scales in Parkinson's Disease: Critique and Recommendations. *Movement Disorders*.

[4] Martinez-Martin P. (1997). A new clinical tool for gait evaluation in Parkinson's disease. *Clinical Neuropharmacology*.

[5] Rubenstein L. M. (1998). The usefulness of the functional status questionnaire and medical outcomes study short form in parkinson's disease research. *Quality of Life Research*.

[6] Weisscher N., Post B., De Haan R. J., Glas C. A. W., Speelman J. D., Vermeulen M. (2007). The AMC Linear Disability Score in patients with newly diagnosed Parkinson disease. *Neurology*.

[7] Gazibara T., Stankovic I., Tomic A. (2013). Validation and cross-cultural adaptation of the Self-Assessment Disability Scale in patients with Parkinson's disease in Serbia. *Journal of Neurology*.

[8] Lang A. E., Eberly S., Goetz C. G. (2013). Movement disorder society unified Parkinson disease rating scale experiences in daily living: Longitudinal changes and correlation with other assessments. *Movement Disorders*.

[9] Martignoni E., Franchignoni F., Pasetti C., Ferriero G., Picco D. (2003). Psychometric properties of the Unified Parkinson's Disease Rating Scale and of the Short Parkinson's Evaluation Scale. *Neurological Sciences*.

[10] Hobson J. P., Edwards N. I., Meara R. J. (2001). The Parkinson's Disease Activities of Daily Living Scale: a new simple and brief subjective measure of disability in Parkinson's disease. *Clinical Rehabilitation*.

[11] Rosqvist K., Hagell P., Odin P., Ekström H., Iwarsson S., Nilsson M. H. (2017). Factors associated with life satisfaction in Parkinson's disease. *Acta Neurologica Scandinavica*.

[12] Brown C. A., Cheng E. M., Hays R. D., Vassar S. D., Vickrey B. G. (2009). SF-36 includes less Parkinson Disease (PD)-targeted content but is more responsive to change than two PD-targeted health-related quality of life measures. *Quality of Life Research*.

[13] Jonasson S. B., Nilsson M. H., Lexell J. (2014). Psychometric properties of four fear of falling rating scales in people with Parkinson's disease. *BMC Geriatrics*.

[14] Olsson Y., Clarén L., Alvariza A., Årestedt K., Hagell P. (2016). Health and social service access among family caregivers of people with Parkinson's disease. *Journal of Parkinson's Disease*.

[15] Lindholm B., Hagell P., Hansson O., Nilsson M. H. (2015). Prediction of falls and/or near falls in people with mild Parkinson's disease. *PLoS ONE*.

[16] Hagell P., Hariz G. M., Nilsson M. H. (2009). P1.131 The Parkinson's disease activities of daily living scale (PADLS) revisited. *Parkinsonism & Related Disorders*.

[17] Hoehn M. M., Yahr M. D. (1967). Parkinsonism: onset, progression and mortality.. *Neurology*.

[18] Fahn S., Elton R. L., Members of the UPDRS Development Committee, Fahn S. Unified Parkinson's Disease Rating Scale. *Recent developments in Parkinson's disease*.

[19] Nilsson M. H., Iwarsson S. (2013). Home and health in people ageing with Parkinson's disease: Study protocol for a prospective longitudinal cohort survey study. *BMC Neurology*.

[20] Kader M., Ullén S., Iwarsson S., Odin P., Nilsson M. H. (2017). Factors contributing to perceived walking difficulties in people with Parkinson's disease. *Journal of Parkinson's Disease*.

[21] Lindholm B., Hagell P., Hansson O., Nilsson M. H. (2014). Factors associated with fear of falling in people with Parkinson's disease. *BMC Neurology*.

[22] Hays R. D., Sherbourne C. D., Mazel R. M. (1993). The RAND 36-Item Health Survey 1.0. *Health Economics*.

[23] Bladh S., Nilsson M. H., Hariz G.-M., Westergren A., Hobart J., Hagell P. (2012). Psychometric performance of a generic walking scale (Walk-12G) in multiple sclerosis and Parkinson's disease. *Journal of Neurology*.

[24] Sheikh J. I., Yesavage J. A. (1986). Geriatric Depression Scale (GDS): recent evidence and development of a shorter version. *Clinical Gerontologist*.

[25] Oswald W. (2005). *Neuropsychological Aging Inventory (NAI) manual*.

[26] Sonn U., Hulter Asberg K. (1991). Assessment of activities of daily living in the elderly. A study of a population of 76-year-olds in Gothenburg, Sweden. *Scandinavian Journal of Rehabilitation Medicine*.

[27] Katz S., Ford A. B., Moskowitz R. W., Jackson B. A., Jaffe M. W. (1963). Studies of illness in the aged. the index of adl: a standardized measure of biological and psychosocial function. *Journal of the American Medical Association*.

[28] Spector W. D., Katz S., Murphy J. B., Fulton J. P. (1987). The hierarchical relationship between activities of daily living and instrumental activities of daily living. *Journal of Chronic Diseases*.

[29] Jakobsson U. (2008). The ADL-staircase: Further validation. *International Journal of Rehabilitation Research*.

[30] Iwarsson S. (1998). Environmental influences on the cumulative structure of instrumental ADL: An example in osteoporosis patients in a Swedish rural district. *Clinical Rehabilitation*.

[31] Iwarsson S., Horstmann V., Oswald F., Wahl H.-W. (2010). Sociocultural care, service context, and IADL dependence among very old European women. *Topics in Geriatric Rehabilitation*.

[32] Nasreddine Z. S., Phillips N. A., Bédirian V. (2005). The Montreal Cognitive Assessment, MoCA: a brief screening tool for mild cognitive impairment. *Journal of the American Geriatrics Society*.

[33] Hobart J., Cano S. (2009). Improving the evaluation of therapeutic interventions in multiple sclerosis: The role of new psychometric methods. *Health Technology Assessment*.

[34] Hobart J., Riazi A., Lamping D., Fitzpatrick R., Thompson A. (2004). Improving the evaluation of therapeutic interventions in multiple sclerosis: development of a patient-based measure of outcome. *Health Technology Assessment*.

[35] Saris-Baglama R. N. (2004). *SF Health Outcomes Scoring Software User's Guide*.

[36] McHorney C. A., Tarlov A. R. (1995). Individual-patient monitoring in clinical practice: are available health status surveys adequate?. *Quality of Life Research*.

[37] Streiner D. L., Norman G. R., Cairney J. (2015). *Health Measurement Scales. A Practical Guide to Their Development and Use*.

[38] de Vet H. C. W. (2011). Measurement in medicine : a practical guide. *Practical guides to biostatistics and epidemiology*.

[39] Nilsson M. H., Hagell P., Iwarsson S. (2015). Psychometric properties of the General Self-Efficacy Scale in Parkinson's disease. *Acta Neurologica Scandinavica*.

[40] Babakus E., Ferguson C. E. (1988). On choosing the appropriate measure of association when analyzing rating scale data. *Journal of the Academy of Marketing Science*.

[41] Carroll J. B. (1961). The nature of the data, or how to choose a correlation coefficient. *Psychometrika*.

[42] Hariz G.-M., Lindberg M., Hariz M. I., Bergenheim A. T. (2003). Does the ADL part of the Unified Parkinson's Disease Rating Scale measure ADL? An evaluation in patients after pallidotomy and thalamic deep brain stimulation. *Movement Disorders*.

